# Cardiovascular Risk Factor and Disease Measures from the Population Assessment of Tobacco and Health (PATH) Study

**DOI:** 10.3390/ijerph18147692

**Published:** 2021-07-20

**Authors:** Martin C. Mahoney, Cheryl Rivard, Hoda T. Hammad, Carlos Blanco, James Sargent, Heather L. Kimmel, Baoguang Wang, Michael J. Halenar, Jueichuan Connie Kang, Nicolette Borek, K. Michael Cummings, Kristin Lauten, Maciej L. Goniewicz, Dorothy Hatsukami, Eva Sharma, Kristie Taylor, Andrew Hyland

**Affiliations:** 1 Roswell Park Comprehensive Cancer Center, Elm & Carlton Streets, Buffalo, NY 14263, USA; Martin.Mahoney@RoswellPark.org (M.C.M.); maciej.goniewicz@roswellpark.org (M.L.G.); Andrew.Hyland@RoswellPark.org (A.H.); 2 U.S. Food and Drug Administration, Center for Tobacco Products, Silver Spring, MD 20993, USA; Hoda.Hammad@fda.hhs.gov (H.T.H.); Baoguang.Wang@fda.hhs.gov (B.W.); Jueichuan.Kang@fda.hhs.gov (J.C.K.); nicolette.borek@fda.hhs.gov (N.B.); 3 National Institute on Drug Abuse, National Institutes of Health, Bethesda, MD 20892, USA; Carlos.Blanco2@nih.gov (C.B.); Heather.Kimmel@nih.gov (H.L.K.); 4 Geisel School of Medicine at Dartmouth, The C. Everette Koop Institute at Dartmouth, Lebanon, NH 03756, USA; James.D.Sargent@dartmouth.edu; 5 Westat, Rockville, MD 20850, USA; MichaelHalenar@westat.com (M.J.H.); KristinLauten@westat.com (K.L.); EvaSharma@westat.com (E.S.); KristieTaylor@westat.com (K.T.); 6 US Public Health Service Commissioned Corps, Silver Spring, MD 20993, USA; 7 Department of Psychiatry & Behavioral Sciences, Medical University of South Carolina, Charleston, SC 29425, USA; CummingK@musc.edu; 8 Medical School, University of Minnesota, Minneapolis, MN 55414, USA; Hatsu001@umn.edu

**Keywords:** survey methods, validity, health behavior, cardiovascular outcomes

## Abstract

Background: Cardiovascular disease is a key health condition associated with tobacco use; however, clinical measures are not typically possible in population-based studies. In this paper, we assess the reliability and validity of self-reported cardiovascular risk factors and diseases in a large nationally representative study of tobacco use and health outcomes. Methods: This paper analyzes self-reported cardiovascular risk factors and disease among adults age 40 years and older based on U.S. nationally representative data from the Population Assessment of Tobacco and Health (PATH) Study. Prevalence of cardiovascular risk factors (self-reported high blood pressure, high cholesterol, diabetes and family history of premature heart disease, BMI ≥ 35, and tobacco use) and cardiovascular disease (self-reported heart attack, stroke and/or congestive heart failure (CHF)) were considered along with ratings of physical functioning, fatigue, and general health. Results: Self-reported cardiovascular disease was found to be associated with functional health measures (walking up a flight of stairs) and general ratings of health. Prospective analyses found strong correlations between sequential data collection waves for history of hypertension, elevated cholesterol and CHF, while more modest correlations were noted for stroke and heart attack. The overall prevalence of cardiovascular disease and hypertension was comparable to those from the National Health and Nutrition Examination Survey (NHANES). Conclusions: These analyses suggest reliability and concurrent validity regarding self-reported cardiovascular risk factors and disease assessed in the PATH Study.

## 1. Introduction

Citing preceding reports from the Office of the Surgeon General, the 2014 Surgeon General’s Report [1] affirmed that cigarette smoking causes coronary heart disease and stroke. This affirmation further extended evidence from the Framingham study identifying smoking as a key risk factor for heart attack, stroke, and congestive heart failure (CHF) [2]. A 1992 American Heart Association Position Statement on Smoking and Cardiovascular Disease for Health Care Professionals described endothelial injury from smoking resulting in atherosclerosis, combined with increased risk of thrombosis, as mechanisms of vascular damage [3]. Additionally, hypertension, elevated cholesterol, obesity, diabetes and a family history of premature heart disease are each well-established risk factors for cardiovascular disease [4,5]. 

Because cardiovascular disease is a key health condition associated with tobacco use, it is important for the Population Assessment of Tobacco and Health (PATH) study to include rigorous and validated measures of cardiovascular health that can be used by the research community. This paper examines reliability and validity for self-reported cardiovascular diseases and compares the prevalence of self-reported cardiovascular diseases and cardiovascular risk factors to the self-reported prevalence rates for the National Health and Nutrition Examination Survey (NHANES) overall and by tobacco use. Reliability of the measures was examined by assessing the extent to which self-reported history of cardiovascular risk factors and diseases at one data collection wave were associated with the same measures at subsequent waves. The validity of the measures was examined by assessing the extent to which cardiovascular risk factors and diseases at one wave were associated with: (1) general health and functional health measures (walking up a flight of stairs, carrying groceries, etc.) assessed at the same wave; and (2) the self-reported prevalence measures for cardiovascular diseases in a similar group in NHANES, among never, former and current cigarette smokers.

Using data from adults age 40 and over who participated in Wave 3 of the PATH Study, this analysis assesses the reliability and validity of self-reported cardiovascular risk factors and diseases, and uses them to estimate the prevalence of these conditions in the general population, and among never, former, and current cigarette smokers. 

## 2. Methods

### Study Design, Setting and Participants

The National Institutes of Health (NIH), through the National Institute on Drug Abuse (NIDA), is partnering with the Food and Drug Administration’s (FDA) Center for Tobacco Products (CTP) to conduct the PATH Study under a contract with Westat. The PATH Study is an ongoing, nationally representative, longitudinal cohort study of adults and youth in the United States (U.S.). The study uses audio computer-assisted self-interviews (ACASI) available in English and Spanish to collect self-reported information on tobacco-use patterns and associated health behaviors.

The PATH Study recruitment for the Wave 1 Cohort employed a stratified address-based, area-probability sampling design that oversampled adult tobacco users, young adults (18 to 24 years), and African-American adults. A nonresponse bias analysis for Wave 1 (found at http://doi.org/10.3886/ICPSR36231, accessed on 15 July 2021) found that the weighted distributions of demographic characteristics for the Wave 1 adult sample are similar to those from the one-year 2013 American Community Survey (ACS) for gender, age and education although there are slightly higher weighted percentages of white, black and Hispanic people in the PATH Study sample compared to ACS. An in-person screener was used at Wave 1 to randomly select youths and adults from households for participation in the study, which included the ACASI interview and, among adults, collection of biospecimens. The PATH study Waves 2 and 3 data collection protocols followed procedures to interview each respondent close to the one-year anniversary of their participation in the prior wave. 

Full-sample and replicate weights were created that adjust for the complex sample design (e.g., oversampling at Wave 1) and nonresponse at Waves 1–3. Because the individuals in the study were selected with the use of a probability sample, the weights allow analyses of the PATH Study data to obtain statistically valid estimates for the U.S. population ages 12 years and older, and the replicate weights enable computation of associated measures of statistical precision.

Weighted estimates from the PATH Study represent the resident population of the U.S. ages 12 years and older at the time the specific data examined were collected (i.e., Wave 1, 2, or 3 who were in the civilian, noninstitutionalized population at Wave 1. This analysis used Wave 3 weights (both single-wave and all-waves) to obtain statistically valid estimates from Wave 3 of the PATH Study.

Further details regarding the PATH Study design and Wave 1 methods are published elsewhere [6,7]. Details on interviewing procedures, questionnaires, sampling, weighting, response rates, and accessing the data are described in the *PATH Study Restricted Use Files User Guide* at https://doi.org/10.3886/Series606 (accessed on 15 July 2021). The study was conducted by Westat and approved by the Westat Institutional Review Board. All respondents ages 18 and older provided informed consent, while youth respondents ages 12 to 17 provided assent and his/her parent/legal guardian provided consent.

Due to the very low prevalence of cardiovascular outcomes in younger adults [4], the current study analyzes data from adults age 40 and over. Cardiovascular outcomes are assessed at Wave 2 (2014–2015; *n* = 12,293), and Wave 3 (2015–2016; *n* = 11,748). Our analyses utilized the adult Restricted-Use Files. Missing data on age, sex, race, Hispanic ethnicity, adult education were imputed as described in the *PATH Study Restricted Use Files User Guide*.

NHANES [8] is an annual study designed to assess the health and nutritional status of adults and children in the U.S. Data are collected on the prevalence of chronic conditions in the population. The current study compares prevalence of cardiovascular conditions from the PATH Study with prevalence from NHANES self-reported data, using NHANES data from 2015–2016. The NHANES survey content from 2015–2016 most closely aligns with the PATH Study instrument content, and as such, the PATH Study data from Wave 3 (2015–2016) are used for this comparison. The self-reported NHANES variables used in this study are cigarette use status, diagnosed CHF, stroke, heart attack, and high blood pressure. For these conditions, previous methodology studies have shown relatively high rates of validation, demonstrating their utility for disease surveillance studies [9,10,11].

## 3. Measures

### 3.1. Tobacco Use Status

Using Wave 3 of the PATH Study, we categorized PATH Study tobacco use status as: non-users (no past 30-day use of tobacco), exclusive cigarette smokers (past 30-day use of cigarettes only), poly-tobacco users (past 30-day use of cigarettes and at least one other tobacco product), and other tobacco users (past 30-day non-cigarette tobacco use). In addition, in order to compare the PATH Study data to NHANES, we categorized the PATH Study cigarette use status as either never (never smoked a cigarette, even one or two puffs), current (ever smoked a cigarette, smoked at least 100 cigarettes in life and now smokes every day or some days), or former cigarette smokers (ever smoked a cigarette, smoked at least 100 cigarettes in life and now do not smoke at all or have not smoked in the past 12 months). Categories of cigarette use for NHANES are never (never smoked a cigarette, even one time), current (smoked at least 100 cigarettes in life and now smokes every day or some days) or former cigarette smokers (smoked at least 100 cigarettes in life and now do not smoke at all).

### 3.2. Cardiovascular Risk Factors

The PATH Study questionnaire included several questions about health and medical history that are known to be risk factors for cardiovascular conditions [12]. Adult participants were asked: “Has a doctor or other health professional ever told you that you had any of the following conditions?’ Responses included high blood pressure, high cholesterol and/or diabetes. Participants were also asked, ‘Were any of your close biological or blood relatives ever told by a health professional that they had a heart attack or needed bypass surgery?’ If yes, ‘Were they told they had a heart attack or needed bypass surgery before the age of 50?’ A response of ‘Yes’ to both of these was used to categorize a participant as having a family history of premature heart disease. Finally, a body mass index (BMI) was calculated for each participant (not including pregnant women) based on their self-reported height and weight. A BMI of 35 or higher was considered to be a health risk. Responses to these items were used to create a health risk score (range 0 [none reported] to 5 [all 5 reported]). The health risk score does not include smoking status since one of the goals of this paper was to develop a measure of cardiovascular disease for the PATH Study data to be used by subsequent papers examining tobacco use as a risk factor for cardiovascular disease, independent of other cardiovascular risk factors. 

### 3.3. Functionally Important Health Measures

Physical functioning, fatigue, and general health were assessed with questions adapted from the Patient Reported Outcomes Measurement Information System (physical question bank) [13] and NHANES [8]. The questions were:

“Does your health limit you in any of the following activities: Walking 3 blocks?” (responses: yes/no),

“In the past 7 days, how would you rate your fatigue on average? By fatigue, we mean feeling unrested or overly tired during the day, no matter how many hours of sleep you’ve had,” (responses: none, mild, moderate, severe, very severe), and “In general, how would you rate your physical health?” (responses: excellent, very good, good, fair, poor).

### 3.4. Cardiovascular Diseases

We developed a dichotomous variable for cardiovascular diseases overall as an outcome. Cardiovascular diseases were measured with a series of questions in which adult participants were asked, “Has a doctor or other health professional ever told you that you had any of the following conditions?” Responses included CHF, stroke, heart attack (also called myocardial infarction) or needed bypass surgery, some other heart condition, none of the above (yes, no). Participants who had ever been told that they had CHF, stroke, or heart attack, were classified as having a cardiovascular disease. 

### 3.5. Covariates

Covariates included variables known to be associated with cardiovascular diseases. These included tobacco use status, sociodemographic variables (age and sex), and health risks (BMI ≥ 35, diabetes, high blood pressure, high cholesterol, and family history of premature heart disease). 

## 4. Statistical Analysis

Analyses using the PATH Study data were weighted using either the Wave 3 single-wave (Table 1 and Table 2) or all-waves (Table 2) full-sample and replicate weights; variances were estimated using the balanced repeated replication (BRR) method [14] with Fay’s adjustment set to 0.3 to increase estimate stability [15]. All analyses were conducted using Stata survey data procedures, version 15.1 (StataCorp LLC, College Station, TX, USA). We first examined weighted prevalence estimates of cardiovascular risk factors and diseases for Wave 3 (Table 1). We then focused on the associations between past 12-month cardiovascular risk factors and diseases at Wave 2 with past 12-month cardiovascular risk factors and diseases at Wave 3 using unweighted Pearson correlations, as well as weighted chi-square tests (Table 3). Finally, we examined the associations between functionally important health measures and risk factors with the presence of cardiovascular disease at Wave 3 using either weighted binary logistic regression or weighted multinomial logistic regression to obtain adjusted odds or risk ratios and 95% confidence intervals (CI) (Table 2).

## 5. Results

Table 1 presents cardiovascular risk factors and diseases among adults age 40+ years in Wave 3 (*n* = 11,748) of the PATH Study. The most common risk factors were high blood pressure and elevated cholesterol; one-quarter of adults have at least one health risk. The most common cardiovascular conditions were heart attack and stroke; the prevalence of having any of the three cardiovascular conditions: heart attack, stroke or CHF, was 9.6%. 

## 6. Reliability and Validity

The reliability of cardiovascular disease measures among 11,079 adults who were age 40+ years at Wave 2 was assessed in Wave 2 and Wave 3. Cardiovascular diseases in the past 12 months at Wave 2 were associated with the same past 12-month diseases at Wave 3. The Pearson correlation between hypertension and elevated cholesterol at Wave 2 and Wave 3 was 0.70 and 0.56, respectively. The correlation between CHF at Wave 2 and Wave 3 was 0.55, while for stroke the correlation was 0.37 and for heart attack the correlation was 0.33 (see Table 3).

Based on data for Wave 3, cardiovascular diseases are associated with difficulty with walking three blocks, greater levels of fatigue and less favorable self-rated physical health (see Table 2). Logistic models determined that adults who have difficulty walking three blocks are more likely to have a cardiovascular disease (odds ratio [OR] 2.90, 95% CI 2.28–3.69) compared to adults who do not have difficulty with walking three blocks; adults with moderate, severe or very severe fatigue are more likely to have a cardiovascular disease compared to adults with no fatigue; adults who rate their physical health as good, fair or poor are more likely to have a cardiovascular disease compared to adults who rate their physical health as excellent (see Table 2). Having one or more cardiovascular risk factors was associated with an increased risk of a cardiovascular disease. 

**Figure 1:**
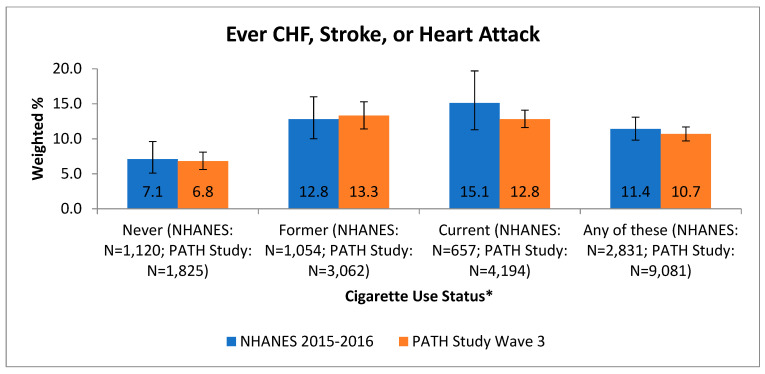
Comparison of PATH Study heart condition prevalence with NHANES self-reported prevalence by tobacco user group and overall, age 40+. * NHANES: never: never smoked a cigarette, even one time; former: smoked at least 100 cigarettes in life and now smokes not at all; current: smoked at least 100 cigarettes in life and now smokes every day or some days. PATH Study: never: never smoked a cigarette, even one or two puffs; former: ever smoked a cigarette, smoked at least 100 cigarettes in life and now smokes not at all or has not smoked in the past 12 months; current: ever smoked a cigarette, smoked at least 100 cigarettes in life and now smokes every day or some days; any of these: respondents who were classified as either never, former, or current cigarette smokers. Note: all Ns are unweighted.

**Figure 2:**
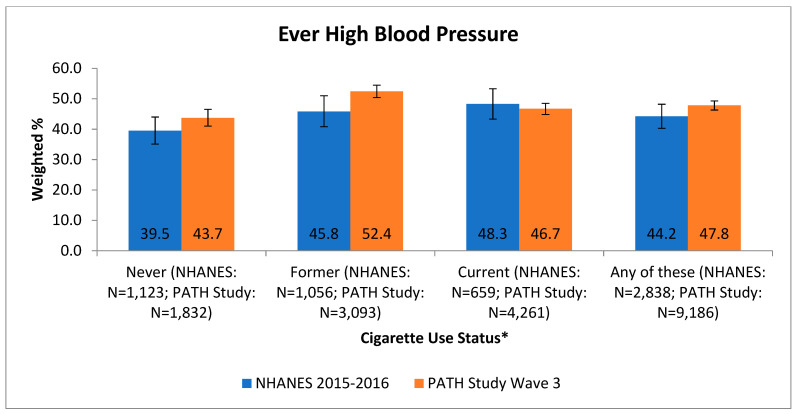
Comparison of PATH Study high blood pressure prevalence with NHANES self-reported prevalence by tobacco user group and overall, age 40+. * NHANES: never: never smoked a cigarette, even one time; former: smoked at least 100 cigarettes in life and now smokes not at all; current: smoked at least 100 cigarettes in life and now smokes every day or some days. PATH Study: never: never smoked a cigarette, even one or two puffs; former: ever smoked a cigarette, smoked at least 100 cigarettes in life and now smokes not at all or has not smoked in the past 12 months; current: ever smoked a cigarette, smoked at least 100 cigarettes in life and now smokes every day or some days. Note: all Ns are unweighted.

NHANES 2015–2016 most closely aligned with the PATH Study data collection for Wave 3 (2015–2016), which occurred during the same time period. As shown in Figure 1, the overall prevalence of a heart condition among persons age 40 years and older, who could be classified as never, former, or current smokers, based on NHANES 2015–2016 (11.4%, 95% CI = 9.8–13.1%) and PATH Study Wave 3 (10.7%, 95% CI = 9.7–11.7%) were comparable. When responses were stratified by smoking status (never, former, or current), they were also comparable (see Figure 1). The never smokers in both data sets had a lower prevalence of any heart condition compared to current or former smokers (see Figure 1). The prevalence of individual cardiovascular conditions (CHF, heart attack, stroke) was comparable between the PATH Study and NHANES (data not shown). As shown in Figure 2, the overall prevalence of high blood pressure among persons age 40 years and older, who could be classified as never, former, or current smokers, was not significantly different in NHANES 2015–2016 (44.2%, 95% CI = 40.3–48.2%) and PATH Study Wave 3 (47.8%, 95% CI = 46.3–49.3%). Prevalence estimates from the two data sets were also similar when stratified by smoking status (see Figure 2).

## 7. Discussion

Cardiovascular disease, including stroke, represents a major cause of morbidity and is the leading cause of death in the U.S. [16] and the strong association between cardiovascular disease and smoking offers a major opportunity for primary and secondary prevention efforts. The earliest evidence for the role of cigarette smoking in lung cancer was identified with case-control studies [17], and further epidemiologic research has concluded that smoking causes at least 15 types of cancer, as well as numerous chronic diseases including heart disease and stroke [18]. The PATH Study was launched to generate longitudinal epidemiologic data on tobacco use behaviors among the U.S. population. Nationally representative surveys, such as NHANES, serve to monitor the prevalence of risk factors, behaviors and disease across the population. The similarity of findings from these analyses with prevalence estimates from NHANES support the validity of the PATH Study measures for cardiovascular disease and cardiovascular risk factors. 

We investigated whether cardiovascular disease was associated with measures of general and functional health. After multivariable adjustment for major cardiovascular disease risk factors, existing cardiovascular disease was associated with difficulty walking three blocks, greater levels of fatigue, and less favorable rating of general physical health. Previous studies have shown that fatigue is commonly reported among those who have suffered a stroke [19,20], and that poor physical functioning and fatigue are related to lower quality of life among cardiovascular disease patients [21]. We noted that risk factor summary score was associated with cardiovascular disease diagnosis as would be expected. However, it was not possible to determine which of these risk factors were identified prior to the cardiovascular disease diagnosis. 

While we did not examine differences by sex, an analyses of NHANES data among adults ages 20–79 from 2001–2002 to 2105–2016 concluded that trends in cardiovascular risk factors were generally similar between males and females [22]. That study reported no changes in the prevalence of hypertension among males or females, a reduced prevalence of smoking among both males and females, reductions in total cholesterol (males > females), increased prevalence of diabetes in both males and females and increased BMI (females > males) during this time period. In addition, there was no substantial change in the total number of risk factors among males or females. 

Several study strengths and limitations warrant mention. These analyses used PATH Study data, a large ongoing longitudinal, nationally representative cohort study. Much of the analyses are focused on Wave 3 given the alignment of data collection period with NHANES 21015–2016. While it is possible that findings over a longer period of time may be different, examination of prevalence data across waves suggests only minimal differences. We also excluded survey participants <40 years of age given the very low prevalence of self-reported cardiovascular disease and typical cardiovascular risk factors among the younger group. It is possible that cardiovascular events in a younger age group may have resulted from unusual clustering of risk factors [23] or even non-standard risk factors. The presence of respiratory conditions, some of which are associated with smoking (e.g., chronic obstructive pulmonary disease) could have impacted the functional outcome measures examined. Finally, we were unable to examine medication lists to validate self-reported diagnosis since all respondents were not systematically asked to provide information on prescribed medications. 

In conclusion, these data support the validity of the PATH Study measures for cardiovascular disease and risk factors. Our analyses from the PATH Study demonstrate that adults with a diagnosis of a heart attack, CHF or stroke also have difficulty walking three blocks, greater levels of fatigue, and less favorable self-ratings of physical health (e.g., concurrent validity). In addition, adults with a cardiovascular condition, such as high blood pressure or elevated cholesterol at Wave 2, were likely to have the same condition or risk factor at Wave 3 (e.g., reliability). Finally, PATH Study data regarding the prevalence of a cardiovascular condition or high blood pressure were comparable to the NHANES data overall and when stratified by tobacco user group for the same data collection period. 

## Figures and Tables

**Table 1:** Cardiovascular Risk Factors and Diseases Among Adults age 40+ in Wave 3 of the PATH Study.

		Wave 3 (*n* = 11,748)		
Risk Factors		N	%	SE
Tobacco use status	Non-users	5951	77.0	0.4
	Exclusive cigarette smokers	3329	12.9	0.3
	Poly-tobacco users	1398	5.2	0.2
	Other tobacco users	1049	5.0	0.2
Selected cardiovascular	High blood pressure ^(b)^	5391	46.7	0.6
risk factors	High cholesterol ^(b)^	4755	43.2	0.7
	Diabetes	3052	26.7	0.7
	Family history of premature heart disease	1761	13.6	0.4
	BMI >= 35	1718	14.2	0.4
	Any of these	8296	72.1	0.5
Number of cardiovascular	0	3151	27.9	0.5
risk factors	1	3231	27.9	0.5
	2	2604	22.8	0.4
	3	1727	15.4	0.5
	4	613	5.0	0.3
	5	121	0.9	0.1
			95% CI	
		mean	lower	upper
Health risk score (0 through 5) ^(a)^		1.4	1.4	1.5
				
Diagnoses		N	%	SE
Congestive heart failure (CHF) ^(b)^		505	3.9	0.2
Stroke ^(b)^		557	4.0	0.3
Heart attack ^(b),(c)^		589	4.8	0.3
Any of these		1221	9.6	0.4
Number of these reported	1	836	6.7	0.4
	2	271	2.1	0.2
	All 3 of these	76	0.5	0.1
Some other heart condition ^(b),(d)^		1480	12.9	0.4

Notes: Unweighted N’s; weighted estimates; SE, standard error ^(a)^ Developed as the sum of health risks ever reported: high blood pressure (yes = 1), high cholesterol (yes = 1), diabetes (yes = 1), family history of premature heart disease (yes = 1), and BMI (35 or higher = 1) ^(b)^ Has a doctor or other health professional ever told you that you had any of the following conditions? (choose all that apply) ^(c)^ Also called myocardial infarction. Option includes ‘needed bypass surgery’ ^(d)^ Respondents were not asked to specify.

**Table 2:** Association between Functionally-Important Health Measures and Cardiovascular Disease, Wave 3 (*n* = 11,748).

Cardiac Condition Outcome ^(a)^ Adjusted Odds Ratio or Relative Risk Ratio ^(b)^																			
Model 1: Walking 3 Blocks ^(c)^					Model 2: Fatigue ^(d)^					Model 3: Physical Health ^(e)^					Model 4: Risk Factor Score ^(f)^				
	OR	SE	Lower	Upper		RR	SE	Lower	Upper		RR	SE	Lower	Upper		OR	SE	Lower	Upper
No	Ref				None	Ref				Excellent	Ref				0	Ref			
Yes	2.90	0.35	2.28	3.69	Mild	1.21	0.18	0.90	1.63	Very good	1.85	0.60	0.98	3.51	1	2.97	0.69	1.88	4.70
					Moderate	1.89	0.26	1.44	2.48	Good	3.52	1.18	1.81	6.83	2	6.58	1.44	4.27	10.15
					Severe	2.73	0.52	1.87	3.99	Fair	7.12	2.30	3.75	13.51	3	10.66	2.47	6.74	16.87
					Very Severe	4.16	1.12	2.44	7.11	Poor	16.83	6.07	8.23	34.42	4	23.01	5.37	14.49	36.55
															5	30.51	10.28	15.64	59.53
Yes	2.90	0.35	2.28	3.69	Mild	1.21	0.18	0.90	1.63	Very good	1.85	0.60	0.98	3.51	1	2.97	0.69	1.88	4.70
					Moderate	1.89	0.26	1.44	2.48	Good	3.52	1.18	1.81	6.83	2	6.58	1.44	4.27	10.15
					Severe	2.73	0.52	1.87	3.99	Fair	7.12	2.30	3.75	13.51	3	10.66	2.47	6.74	16.87
					Very Severe	4.16	1.12	2.44	7.11	Poor	16.83	6.07	8.23	34.42	4	23.01	5.37	14.49	36.55
															5	30.51	10.28	15.64	59.53
Yes	2.90	0.35	2.28	3.69	Mild	1.21	0.18	0.90	1.63	Very good	1.85	0.60	0.98	3.51	1	2.97	0.69	1.88	4.70

Note: ^(a)^ Has a doctor or other health professional ever told you that you had any of the following conditions? Choose all that apply: congestive heart failure, stroke, heart attack (no, yes: the outcome reported here is a ‘yes’ response to any of these three conditions) ^(b)^ Models 1-3 control for age, gender, body mass index, self-report of any of the following: diabetes, cancer, high blood pressure, high cholesterol, family history of premature heart disease, and cigarette use status. Model 4, risk factor score, controls for age, gender, and cigarette use status. ^(c)^ Does your health limit you in doing any of the following activities? Walking 3 blocks (no, yes); *n* = 10,664. ^(d)^ In the past 7 days, how would you rate your fatigue on average? By fatigue, we mean feeling unrested or overly tired duing the day, no matter how many hours of sleep you’ve had. (none, mild, moderate, severe, very severe); *n* = 10,666. ^(e)^ In general, how would you rate your physical health? (excellent, very good, good, fair, poor); *n* = 10,675. ^(f)^ Developed as the sum of risk factors ever reported: high blood pressure (yes = 1), high cholesterol (yes = 1), diabetes (yes = 1), family history of premature heart disease (yes = 1), and BMI (35 or higher = 1); *n* = 11,170.

**Table 3:** Reliability of Cardiovascular Risk Factor and Disease Measures among 11,079 Adults who were Aged 40+ at Wave 2.

Condition Reported Past 12 Months at Wave 2			Condition Reported Past 12 Months at Wave 3			Pearson Correlation Coefficient (Unweighted)	Chi Square *p*-Value (Weighted)
			Yes (N)	Yes (%)	SE		
High Blood Pressure	Yes	3666	2961	80.8	0.9	0.70	0.00
	No	7323	795	10.3	0.4		
High Cholesterol	Yes	2587	1740	67.4	1.2	0.56	0.00
	No	8402	915	11.5	0.4		
Congestive Heart Failure	Yes	254	150	56.0	4.8	0.55	0.00
	No	10735	129	1.1	0.1		
Stroke	Yes	174	69	44.9	5.2	0.37	0.00
	No	10815	124	1.0	0.1		
Heart Attack	Yes	166	57	35.8	6.1	0.33	0.00
	No	10823	113	1.0	0.1		
Some other heart condition	Yes	604	280	49.7	2.6	0.42	0.00
	No	10385	364	3.6	0.2		
